# I Quit! Effects of Work-Family Policies on the Turnover Intention

**DOI:** 10.3390/ijerph18041893

**Published:** 2021-02-16

**Authors:** José Aurelio Medina-Garrido, José María Biedma-Ferrer, María Vanessa Rodríguez-Cornejo

**Affiliations:** INDESS, Universidad de Cádiz, 11405 Jerez de la Frontera, Spain; josemaria.biedma@uca.es (J.M.B.-F.); vanesa.rodriguez@uca.es (M.V.R.-C.)

**Keywords:** work-family balance, work-family policies, well-being, turnover, structural equation modelling, SEM

## Abstract

The retention of key human resources is a challenge and a necessity for any organisation. This paper analyses the impact of the existence and accessibility of work-family policies on the well-being of workers and their intention to leave the organisation. To test the proposed hypotheses, we applied a structural equation model based on the partial least squares path modelling (PLS-SEM) approach to a sample of 558 service sector workers. The results show that the existence and accessibility of work-family policies directly reduce the intention to leave the organisation. Moreover, this relationship also occurs indirectly, by mediating the well-being that is generated by these work-family policies. We also analysed the moderating role that gender and hierarchy could have in the above relationships. In addition to the above theoretical implications, this study has practical implications. The findings show that employees with family and work balance problems experience lower emotional well-being, more health problems and eventually higher turnover rates. To avoid these problems, management must focus not only on the implementation of work-family policies but also on their accessibility, without subsequent retaliation or prejudice to employees. Additionally, management should pay special attention to female managers, given their greater difficulty in balancing work and family life.

## 1. Introduction

Experienced and well-trained professionals who leave their organisations are a major problem for management [1]. Retaining key employees, who are fundamental to the growth and development of organisations, is both a challenge and a necessity [2]. Managing the turnover intention of these employees is vital to the survival of organisations, especially in the service industry [3], since high turnover rates can mean high costs [4] and organisations lose the investments made in training, development and retention when valuable employees leave. In addition, it is necessary to retrain new employees to replace those who have left [5]. For this reason, some human resource management practices aim at retaining workers by reducing their turnover intention [6]. These practices include work-family policies (WFPs). WFPs are human resources practices that organisations implement to promote and facilitate work-family balance [7]. Some examples of WFPs would be flexible working hours, teleworking, breastfeeding leave, and financial support for childcare [8].

The determinants of turnover intention include work-related stress [9,10], job dissatisfaction [11,12,13], harassment at work [9,14], burnout syndrome [15], lack of commitment to the organisation [16], and work-family conflict (WFC) [17,18]. The WFC arises when it is difficult to balance workers’ work and personal lives [19]. This difficulty, coupled with the ongoing demand for improved outcomes, puts strong psychological pressures on workers [4]. As a result, the WFC can lead to job stress, negative performance effects and worker turnover [20].

Organisations aim to reduce the impact of WFC by implementing WFPs that mitigate it [21] and ultimately prevent job abandonment. The implementation of WFPs reduces work stress [22], absenteeism [23], and turnover intention [24]. In addition, WFPs improve job satisfaction [25], quality of life [26,27], and workers’ emotional commitment [28,29]. WFPs also have the virtue of reducing the negative effects of WFC on the psychological and physical well-being of workers [30]. For this reason, well-being can act as a mediator between WFPs and other variables such as work performance [8,31], absenteeism [23], and, predictably, turnover intention.

Nevertheless, the mere existence of WFPs does not imply that employees can enjoy these policies without inconvenience [32]. For workers to be able to access WFPs without problems, they must be aware of them [33] and perceive that they can use them freely without retaliation [23,31]. The concept of WFP accessibility is the degree of freedom perceived by workers to use the WFPs offered by their organisation without undermining their career, working conditions, social and professional respect and income [23].

The literature sufficiently recognises the relationship between WFC and turnover [17,18]. However, there is a knowledge gap in the study of the impact of WFPs [24]. In addition, the separate analysis of the relationships of the existence and accessibility of WFPs with turnover and the mediating role of employees’ well-being in these relationships are also novel. In this regard, the aim of the present study is to analyse the impact that the existence and accessibility of WFPs have on turnover intention while considering the mediating effect that well-being has on this relationship. To achieve this objective, we tested these relationships using a structural equation model based on the partial least squares path modelling (PLS-SEM) approach to a sample of 558 service sector workers. The study of WFC and the implementation of WFPs deserves special attention in the service sector [34], where workers commonly suffer from work-related stress [35], which ultimately increases WFC and turnover prevalence.

The results of this study provide, as an added value to the literature on WFPs, a separate analysis of the effect that the existence and accessibility of these policies have on the well-being and turnover intention. In addition, we study how the emotional and physical well-being of employees influences turnover rates. Finally, we analyse the moderating role that gender and hierarchy might have in the above relationships. The interest in studying gender and hierarchy as moderating variables is supported by works that recommend implementing WFPs to avoid WFC for women with responsibilities in the organisation [36]. Similarly, Sharabi [37] adds the importance of reducing WFC for women managers through specific WFPs, such as flexitime and teleworking.

This paper is structured as follows. Section two sets out the theoretical framework that supports the relationships between the existence and accessibility of WFPs, well-being and turnover intention. Section three describes the methodology that has been developed, in that we define the variables and their measures, and apply a structural equation model to the data that were collected from a service sector sample. In section four, the results show that all of the model assumptions are congruent with the postulated sign and all are supported. Furthermore, we found that the mediating effects of emotional and physical well-being are significant. This section also studies the moderating role of gender and the hierarchy. Finally, we discuss and conclude the results of the study.

## 2. Background and Hypotheses

In this section, we present the theoretical foundations of the model of the effect of WFPs on turnover intention, mediated by well-being. The development of this model provides the hypotheses under study.

### 2.1. WFPs and Turnover

According to Role Theory, job satisfaction reduces when there are work and family conflict [38] and this negative relationship has been confirmed empirically by several studies [39,40], with inter-role conflict being considered as a predictor of the level of job satisfaction [41]. More specifically, work interference in the family reduces the level of employees’ satisfaction [42] and this conflicting situation encourages employees to leave their organisations [39,40]. This phenomenon is even more pronounced in the case of long weekly working hours [43]. In this regard, studies consider WFC to be a significant predictor of turnover intention [20].

The WFPs that organisations implement to reduce WFC increase employee commitment to their organisations [44], thus decreasing the intention to leave [45]. Notwithstanding, the literature with studies of WFPs based on appraisal and coping theory [46] indicates that WFPs have a positive correlation with job satisfaction, and reduce stress, deliberate wrong task performance and even abandonment of work [47,48]. Therefore, solving the WFC contributes to increasing job satisfaction and reducing the probability of employees leaving their jobs [49]. In addition, the existence of WFPs increases workers’ loyalty and commitment to their organisations [50], which also reduces turnover rates [51].

However, WFPs must be properly implemented if they are to definitively avoid certain behaviours that are counterproductive to the organisation and to a lesser degree, avoid abandonment [52]. Although WFPs can help reduce turnover, the mere existence of WFPs is not enough. To the best of our knowledge, previous research often forgets that these WFPs must also be accessible to the worker. For WFPs to generate the expected advantages, workers should know that they exist [53], but they should also perceive that WFPs are accessible without retaliation or adverse consequences for their careers [54]. Otherwise, workers may not use WFPs for fear of a perceived lack of commitment to the organisation, losing promotion opportunities or even their jobs [31]. Since the mere existence of WFPs is not sufficient [55], we should separately consider whether workers are aware of the existence of WFPs and whether they also perceive that they are accessible in practice.

On the basis of all the above arguments, we propose two hypotheses:

**Hypothesis** **1** **(H1).** *The existence of WFPs is negatively related to turnover intention.*

**Hypothesis** **2** **(H2).** *The accessibility of WFPs is negatively related to turnover intention.*

### 2.2. WFPs and Emotional Well-Being

WFC is a stress factor, as work responsibilities interfere with employees’ family responsibilities when they are forced to spend more time and energy on their work than on their family [56]. As argued by Role Conflict Theory [57], an individual’s time and energy are limited. The resources spent on the employee’s role exhaust the resources that are available for the family role, thus creating conflict [58]. In this regard, previous studies in the literature associate WFC with burnout and psychological stress [59,60], which in turn reduces the emotional well-being of the worker [61]. On the other hand, WFPs can generate emotional well-being [8,31,62,63,64], since reducing WFC would have positive effects on people’s happiness [65] and would decrease negative emotions [30]. Further, lack of, or difficulty in accessing, WFPs is related to mental health issues such as anxiety and depression [8,23,31]. Based on these arguments, we establish the following hypotheses:

**Hypothesis** **3** **(H3).** *The existence of WFPs is positively related to the emotional well-being of employees.*

**Hypothesis** **4** **(H4).** *The accessibility of WFPs is positively related to the emotional well-being of employees.*

### 2.3. Emotional Well-Being and Physical Well-Being

Emotional and psychological problems can seriously affect health [66,67]. According to Gong et al. [68], problems at work such as stress and burnout that are maintained over time can eventually lead to psychological problems. Moreover, the type of work and the extension of the working day reduces or even eliminates physical activity, both inside and outside the workplace [69] generating adverse effects such as disorders of the musculoskeletal system [70], obesity [71] and diabetes [72]. In this regard, inadequate management causes some of the workers’ illnesses, as management demands on workers can lead to increased work-related and personal stress [73]. Further, stress, in turn, causes psychosocial and physical problems, leading to a deterioration in workers’ health [74].

Based on the above, we propose the following hypothesis:

**Hypothesis** **5** **(H5).** *Emotional well-being is positively related to physical well-being.*

### 2.4. Physical Well-Being and Turnover Intention

Stress, anxiety and a lack of emotional well-being can lead to physical exhaustion and other physical problems, such as nausea, muscular pain, and cardiovascular disease, among others [60,74,75,76,77]. These health problems and lack of physical well-being reduce workers’ performance and increase their possible intention to leave the organisation [76,77,78].

Health problems and general physical discomfort can lead to functional difficulties, lack of capacity for adequate performance or even sick leave for workers. Nevertheless, one of the most common side effects of the lack of physical well-being is a decrease in employee motivation and organisational commitment, factors that increase turnover intention [79]. For this reason, retaining employees means improving motivation, which in turn requires improving their physical well-being [80,81].

The above arguments lead to the following hypothesis:

**Hypothesis** **6** **(H6).** *Physical well-being of the worker is negatively related to turnover intention.*

### 2.5. Gender and Hierarchy Moderating Effect

The literature on the WFC shows that women experience more difficulty than men in balancing [82,83]. Working women carry a double burden as employees, as housewives and as primary caregivers [84,85]. In this regard, WFC is higher in women than in men as a result of the overload of family care, specifically for children and the elderly [86]. The work-life balance is more complex for women, as they find it harder to ignore family problems, especially children’s concerns [87,88]. The previous literature on women’s dual roles predicts that the existence and accessibility of WFPs will be more crucial to women, so WFPs impact on well-being and turnover intention may be different from that of men. However, there is also evidence that gender does not influence turnover intention [89]. These contradictory results could be the case in some situations because of the influence of cultural and sectoral determinants on this phenomenon [8,31].

However, the impact of WFPs may also depend on the position in the organisational hierarchy. In this regard, the greater the responsibility at work, the higher the level of WFC a worker might experience [90,91]. Thus, being a woman and a manager is a double challenge, which makes it particularly complex to balance work and family life [36].

**Figure 1:**
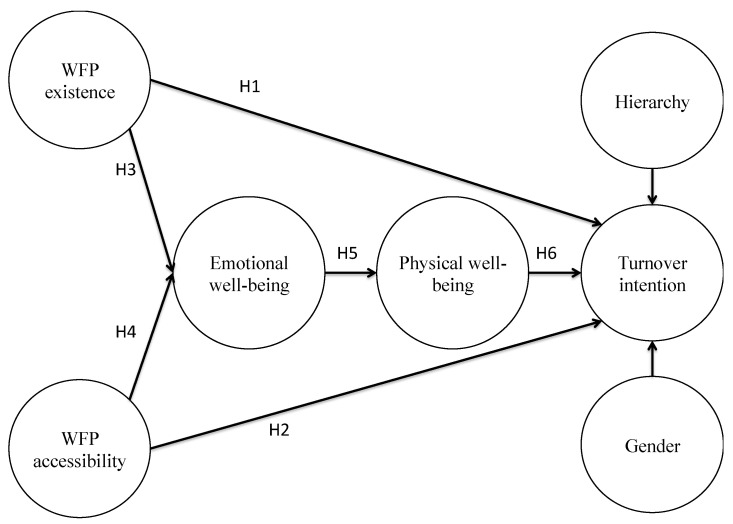
Theoretical model and hypotheses.

Given the relevance of this research question, this paper considers the moderating role that gender and the hierarchy might have in the above hypotheses. Considering the relations previously established in the hypotheses and the moderating role of gender and hierarchy noted above, Figure 1 depicts the theoretical model proposed for verification.

## 3. Methodology

### 3.1. Sample

The sample obtained was made up of 584 workers from the Spanish service sector. We collected data by conducting a self-administered questionnaire survey. We emailed the participants a link to an online form. Managers collaborated in the survey process, prescribing their employees to participate and allowing them to complete the questionnaires during working hours. Considering the large number of emails sent, the response rate was 14.7%. The number of participating companies was thirteen. These companies included financial companies, IT companies, insurance companies, contact centres and, to a lesser extent, engineering companies, healthcare companies, retail companies and companies in the tourism sector.

Applying the pre-evaluation of the data proposed by Hair et al. [92], we removed 26 observations due to their high percentage of missing data. Therefore, we carried out the study with 558 valid questionnaires. Of the total number of respondents, 45.3% were women, of whom 16.1% were managers, 68.0% had university studies, and the average age was 44.8 years. Other relevant characteristics that describe the sample are that 90.4% had a partner, 78.5% of whom both worked; 74.1% had a child, and 47.1% had two or more children; 23.5% had a dependent ascendant, and 5.2% had a disabled dependant. Considering the time worked, 18.1% had less than ten years of experience in the company, 42.3% had less than 20 years, and the remaining 39.6% had more than 20 years. Most of the contracts were stable, with only 3.4% being temporary. In terms of average weekly working hours, only 9.6% reported working up to a maximum of 30 h, 55.8% worked an average of up to 40 h, and 34.6% more than 40 h per week. Concerning working shifts, 51.1% worked only morning shifts, 39.7% worked morning and afternoon shifts, and the remaining 9.2% worked afternoon or night shifts, or variable shifts.

We divided the sample into four subsamples to analyse the interaction effect of gender and hierarchy variables, namely: (1) female managers, (2) female employees, (3) male managers, and (4) male employees, composed of 34, 219, 56 and 249 individuals, respectively.

Given that the statistical power of the sample is 0.8 and that the default alpha level is 0.05, we can say that the sample (*n* = 558) made it possible to detect very small effect sizes, which were difficult to identify [93].

### 3.2. Measures

We modelled the variables included in the theoretical model as composites, since they were design variables formed by the linear combination of the indicators. In addition, since the indicators were correlated, we designed all the variables as composite in mode A, using the correlation weights [94].

We used various indicators that assessed the degree of agreement of respondents with various statements using a 7-point Likert scale. These indicators or items were intended to reflect the subjective assessment made by the respondents of the variables of the theoretical model of this research.

We defined the variable existence of WFPs as the subjective perception of individuals that WFPs exist in their organisation. To measure the existence of WFPs, we adopted five items from the Families and Work Institute [95,96] scale, namely: (Regarding WFPs) (1) “Your organisation offers them”; (2) “Your organisation reports on them”; (3) “Know what they consist of”; (4) “You have ever used them”; and (5) “Know employees who have used them”.

As aforementioned, the concept of WFP accessibility is the degree of freedom perceived by individuals to use the WFPs offered by their organisation without undermining their career, working conditions, social and professional respect and income. The accessibility of WFPs combined the contributions of Anderson, Coffey and Byerly [97] and the Families and Work Institute [95,96] into fifteen items, that were: (Considering the use of WFPs) (1*) “The application procedures are simple”; (2) “There is an unwritten rule in my job that an employee cannot attend to family or personal needs during work hours”; (3) “At my workplace, employees who put their family or personal needs ahead of their jobs are not well regarded”; (4) “If you have a problem combining your work responsibilities with family and personal ones, the attitude in my work is: It was your decision, now accept the consequences”; (5*) “When I am offered more hours than stipulated in the employment contract, I can refuse to work them without negative consequences at work”; (6) “In my workplace, employees have to choose between advancing their careers or devoting their attention to their personal or family lives”; (7) “In my workplace, employees who apply for permits or licenses for family or personal reasons, or who agree to different schedules for personal or family needs, are less likely to advance in their jobs or careers”; (8*) “I have the flexible working hours I need at work to manage my personal and family responsibilities”; (If I were to apply to use WFPS...) (9*) “My work responsibilities do not allow it”; (10) “There would be negative consequences regarding advancing my professional career”; (11) “There would be negative consequences regarding my current or future income”; (12) “My superior would not support it”; (13) “My colleagues would not support it”; (14*) It would mean that others would have to do more work; (15) “They would make me appear to be less committed to my job or career”. Subsequently, we removed from the measurement scale the items having a number marked with an asterisk (*).

We defined emotional well-being as a positive psychological, affective and mental health state of individuals. We measured the emotional well-being variable by integrating Warr [98] and Kossek et al. [99] scales into a reflective fifteen-item scale, which was: (“During the last few weeks, I’ve felt all the time....”) (1) “Tense”; (2) “Uneasy”; (3) “Preoccupied”; (4) “Calm”; (5) “Content”; (6) “Relaxed”; (7) “Depressed”; (8) “Sad”; (9) “Unhappy”; (10) “Cheerful”; (11) “Enthusiastic”; (12) “Optimistic”; (13) “Angry”; (14) “Annoyed”; and (15) “Irritated”.

We defined individuals’ physical well-being as the absence of diseases and the development of behaviours to prevent them from having a healthy lifestyle and sufficient energy to fulfil their obligations at work. To measure physical well-being, we used a nine-item scale from the study of Kossek et al. [99], namely: (“During the last few weeks, I’ve felt all the time....”) (1) “Annoying trembling of my hands”; (2) “Shortness of breath when not working physically or hard”; (3) “Pounding heart”; (4) “Faster-than-normal pounding of my heart”; (5) “Sweat on my hands, feeling wet and sticky”; (6) “Momentary dizziness”; (7) “Stomach ache or upset”; (8) “Loss of appetite”; and (9) “Problems with sleeping at night”.

Turnover intention measures whether an organisation’s employees plan to leave their jobs or whether that organisation plans to remove employees from their jobs. In this paper, we only considered employee turnover intention. We measured turnover intention with a scale of eight items from the work of Boshoff and Mels [100], Becker [101], and De Cuyper, Mauno, Kinnunen, and Mäkikangas [102], that was: (1) “I will probably actively look for another job soon”; (2) “I often think about resigning”; (3) “It would not take much to make me resign”; and (4) “There is not too much to be gained by sticking with the organisation indefinitely”.

Finally, we measured the moderating variables of gender (male/female) and hierarchy (worker/manager) dichotomously. In the case of the hierarchy variable, we considered managers to be those who held positions of responsibility or who were in charge of other workers.

### 3.3. Structural Equation Modelling

We proposed a SEM model based on the aforementioned PLS-SEM approach, to test the hypotheses. We followed the methodological guidelines of Hair Jr, Sarstedt, Hopkins, and Kuppelwieser [103] to provide consistent estimations as we have variables modelled as composites. In this analysis, we used SmartPLS software (SmartPLS GmbH, Bönningstedt, Germany) [104].

We evaluated the model by following these steps: assessment of the global model, assessment of the measurement model, assessment of the structural model and finally, analysis of the moderating effect.

### 3.4. Common Method Bias

Common method bias, or common-method variance, is a false variance caused by measuring variables using the same method or source [105]. To study this problem, we used the full collinearity test based on variance inflation factors (VIF) following Kock [105]. According to this test, a VIF value higher than 3.3 would mean that there is a pathological co-linearity, which would imply that the common method bias contaminates the model. In our study, the highest value was 1.54, hence the model was free of bias.

## 4. Results

### 4.1. Assessment of the Global Model

The goodness of the global model fit was the first step for the assessment of the model. If the model did not fit the data, the estimates obtained may be without meaning and therefore the conclusions may be opened to dispute [106]. We made two assessments of the global model. The first assessment considered all of the indicators in the model before the assessment of the measurement model. The second assessment followed the measurement model study and the removal of indicators that did not fulfil the requirements.

For the analysis of the global model, we used the approximate adjustment measures [106]. To be precise, we analysed the standardised root mean squared residual (SRMR), whose threshold was 0.8 [107]. The results showed values of 0.074 before the removal of the indicators and 0.060 after the removal of these indicators. Therefore, the model was better after the removal of the indicators which presented reliability and validity problems. In conclusion, we were able to state that we had an approximately true model.

### 4.2. Evaluation of the Measurement Model

Since the model contained composites estimated in Mode A, we analysed the reliability and validity. The reliability analysis checks that the indicators measured what they should measure, while the validity analysis verified that the measurement was stable and consistent.

Firstly, an analysis of the individual reliability of the item was done, in which a great quantity of the indicators with their respective constructs was examined. We considered the threshold proposed by Carmines and Zeller [108], which accepts items with loads higher than 0.707. Notwithstanding, some researchers suggest not eliminating items with loads between 0.4 and 0.707, in case they do not formulate problems for the rest of the stages of the measurement model [109]. We had to remove three items corresponding to the accessibility of WFPs with very low loads (below 0.4). Subsequently, we removed two more items in this variable to fulfil the requirement for convergent validity. As a result, we managed to increase the value of the average variance extracted (AVE). As for the turnover intention variable, we eliminated four indicators with very low loads.

Secondly, we closely studied the reliability of the construct in order to determine if the items measuring a construct were similar in their scores. For this purpose, we considered the measures corresponding to composite reliability [110] and Dijkstra-Henseler [111]. Nunnally and Bernstein [112] suggest values higher than 0.8 for more advanced stages of research.

Subsequently, we studied the convergent validity to check that the indicators represented a single subjacent construct. This occurred when the AVE had values above 0.5 [113].

Finally, we verified discriminant validity, i.e., the degree to which a given construct was different from other constructs. Table 1 shows this check utilising the HTMT ratio [114]. We also checked this discriminant validity by employing the criterion of Fornell and Larcker [113], using the matrix of correlations between variables.

Once we had removed the items described above, the model met the necessary reliability and validity requirements. Table 1 and Table 2 reflect these results. Correlation weights indicated the contribution of each indicator to the composite. The weights allowed us to check the reliability of the item in the model. Although we noted that one of the weights did not reach the minimum required threshold (λ >= 0.707), the construct reliability (Composite reliability and Dijkstra-Henseler’s (ρA)) was greater than 0.7 and the convergent validity (AVE) was greater than 0.5 in the corresponding latent variable, hence it was not necessary to remove it.

### 4.3. Structural model

**Figure 2:**
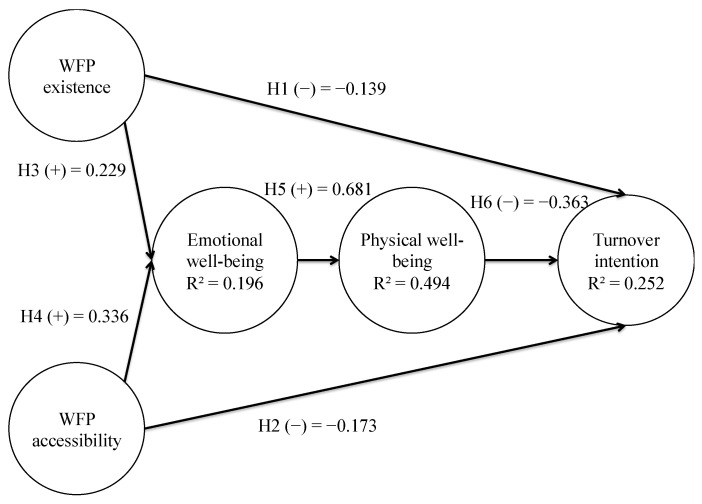
Direct effects. Diagram.

For the assessment of the structural model, we studied closely the relationships raised in the model utilising the bootstrap method with 5000 samples. Along these lines, we assessed the magnitude, sign and significance of the relationships between the variables. In addition, we analysed the model predictive power by means of the coefficient of determination (R²) of the endogenous variables and the decomposition of the explained variance. This permitted us to know the relevance of each of the antecedent variables in the dependent variable. Finally, we used the rules from Cohen [93] to determine the size of the effects. These outcomes are shown in Table 3 and Figure 2.

**Figure 3:**
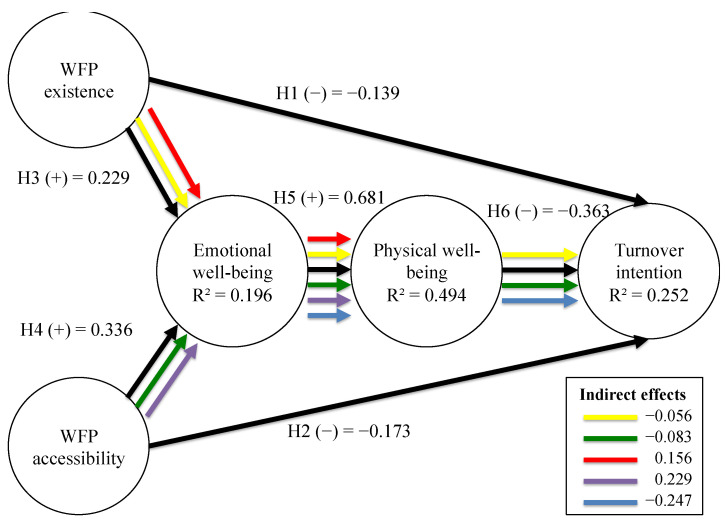
Indirect effects. Diagram.

The R² values obtained in this model indicated a low predictive power for the dependent variables emotional well-being and turnover intention, and a moderate predictive power for the variable physical well-being. The postulated sign made all the relationships of the theoretical model congruent, supported with a small effect for hypotheses H1, H2, H3 and H4, and with a moderate effect for hypothesis H6 and a large effect for hypothesis H5. Finally, we could prove that the mediating effects of the model were significant. Therefore, we could conclude that the variables emotional well-being and physical well-being mediate within the proposed model (Table 4 and Figure 3).

### 4.4. Moderating Effect

Finally, we tested the interaction effect of the moderating variable associated with gender and hierarchy by conducting a multi-group analysis. For this purpose, a division of the sample into four different groups was made: female managers, female employees, male managers and male employees. Firstly, we analysed the invariance of the measure to make sure that the parameters of the measurement model were not the reason for these differences, but rather the reason was linked to the path coefficients. We found partial invariance in two cases, total invariance in two other cases and in the further two cases the invariance of the measure was not met (Table 5). Therefore, for the latter cases, we could not apply the permutation analysis that was developed by Chin [115] to evaluate whether there were significant differences between each pair of groups. We carried out the permutation analysis in the four cases where there was invariance of the measure (Table 6), and we realised that there were only significant differences in the WFPs’ existence and emotional well-being relationship, for the groups “female managers” and “female employees”, and the groups “female managers” and “male employee”.

## 5. Discussion

The findings of this paper have theoretical implications with respect to the body of the literature and also practical implications for business management.

### 5.1. Theoretical Implications

Regarding the theoretical implications, the results obtained reinforce and update the existing evidence in the literature about the negative relationship between WFPs and turnover [51]. In fact, these results are coherent with that part of the literature which proves the relationship between WFC and stress, performance and turnover [20]. In contrast, some authors do not see a direct or indirect relationship between WFC and turnover [116]. If this were the case, the implementation of WFPs to retain valuable staff would not be sufficiently justified. However, our findings are aligned with the literature that does recognise the negative relationship between WFPs and turnover.

In this paper, we analyse the accessibility and existence of WFPs separately. This differentiation allows us to study the impact of these two variables on turnover intention independently. This differentiation adds further value to the literature on WFPs. The results of our work consider that the mere existence of WFPs does not imply a decrease in turnover intention. In addition to the existence of WFPs, these WFPs must be accessible to workers without subsequent retaliation, or detriment to their economic incentives or professional career.

The results of this work also indicate positive relationships between the existence and accessibility of WFPs and the emotional well-being of employees. This relationship is consistent with the literature on the effects of WFPs on well-being [61]. Notwithstanding, these findings reinforce those provided by Medina-Garrido et al. [23]. As with those authors, we confirm that the mere existence of WFPs is not enough to have a positive impact on workers’ emotional well-being, but it is also necessary that these WFPs are accessible to workers.

Our research also corroborates that there is a significant relationship between emotional well-being and physical well-being of employees and that these two variables mediate between WFPs and turnover intention. In line with the literature, these findings confirm the negative relationship between well-being and turnover [117,118]. As examples in the literature point out, in practice, the absence of adequate WFPs can generate WFC, thus decreasing emotional well-being. This may lead to stress and anxiety in workers [119] and subsequently to health problems [60,74,77], thus contributing to increased turnover intention [76,77].

Regarding the moderating variables of gender and hierarchy, we observed significant differences in the existence of WFPs and the emotional well-being relationship between the “female manager” and “female employees” groups, and also between the “female managers” and “male employees” groups. The literature on WFPs does not describe these differences, hence they add value to the previous literature. Moreover, this result is consistent with the literature that generally argues on the particular difficulty female managers have in balancing family and work-life [36]. This result seems to suggest that female managers have more difficulties than other groups in achieving adequate emotional well-being.

### 5.2. Practical Implications for Management

The results of our study also have practical implications for management. If the managers of an organisation want to improve the emotional well-being of their workers and reduce WFC-motivated turnover, then the introduction of WFPs would be a necessary but not sufficient condition. Complementarily, employees should have access to WFPs without reprisals of any kind. Their possibilities of promotion within the organisation and their financial incentives should not be threatened. In this regard, the organisation should show its explicit support for the balance of family and personal life with working life. To this end, the organisation should disseminate a culture of acceptance of work-life balance which prevents social sanctions that would imply disapproval by colleagues and supervisors. We recommend carefully planning the process of implementing WFPs, starting with their proper selection. The WFPs that management should pay more attention to are those that are most valued by employees as a means of balancing work and family. In our research, the most valued measures were the following, in this order: flexible working hours, reduced working hours for child and family care, breastfeeding leave hours, personal leave days, long-term leave for family illness, leave for hospitalisation of a partner or relative, leave to accompany a family member for medical care, long-term leave to care for family and children, and transfer to the work centre closest to the family home.

After implementing the WFPs, the management should monitor that they are working correctly and that workers can access those WFPs without any inconvenience. In this regard, we suggest that management should establish a balanced scorecard, or similar monitoring tool, to compare the implementation objectives and the effective use of each practice. Periodic monitoring of this balanced score-card will ensure the correct functioning and use of the practices or highlight the need to intervene to correct any deviations.

In addition, given the vital role of well-being in reducing turnover intention, managers should be aware that employees with work-life balance problems may suffer from health problems, besides feeling lower emotional well-being. Managers should realise that turnover intention can increase not only because of employees’ difficulties in balancing family and work but also because they can subsequently suffer from stress, anxiety and even physical health problems.

Finally, managers should consider, for the specific case of female managers, the moderating role of gender and hierarchy in the relationship between existing WFPs and emotional well-being. We recommend that organisations pay special attention to female managers. This group experiences greater difficulty in balancing, compared to other groups. Moreover, female managers may have less emotional well-being. This is because they have to balance family responsibilities with management responsibilities. The latter is more demanding responsibilities with a higher level of stress and commitment than for other employees.

## 6. Conclusions

Retaining experienced and well-trained employees is a challenge and a vital need for many organisations [2] but a major determinant of job abandonment is WFC [20], hence reducing this conflict requires organisations to implement WFPs.

This paper aims to analyse the impact that WFPs have on reducing turnover intention. To achieve this objective, we propose a model in which the existence and accessibility of WFPs are negatively related to turnover intention, mediated sequentially by emotional well-being and physical well-being and moderated by gender and hierarchy. We contrast these relationships using a structural equation model based on the PLS-SEM approach to a sample of 558 service sector workers. As a result, all of the proposed relationships are verified, except in the case of moderation, where we only see significant differences between some groups in the relationship between the WFPs’ existence and emotional well-being.

The findings of this paper have theoretical implications for the literature and also practical implications for business management. From a theoretical point of view, we confirm that WFPs and well-being have a negative relationship with turnover intention. Furthermore, we found that both the existence of WFPs and their accessibility have an impact on emotional well-being and turnover intention. We also corroborate that there is a significant relationship between emotional well-being and physical well-being of employees and that these two variables mediate the relationship between WFPs and turnover intention. Finally, we found significant differences in the WFPs’ existence and emotional well-being relationship between “female managers” and “female employees” groups and also between “female managers” and “male employees” groups.

These theoretical findings have important practical implications. Given the results obtained, we recommend that managers take care not only of the implementation of WFPs but also of their accessibility. Regarding the implementation of WFPs, management should pay more attention to the WFPs that are most valued by employees to balance work and family. Once WFPs are in place, management should monitor that they are working correctly (e.g., using a balanced score-card or similar monitoring tool). Concerning the accessibility of WFPs, workers should perceive that the use of WFPs does not lead to retaliation or further damage. Managers should show their express support for the use of such WFPs and spread a culture of acceptance of work-life balance. In addition, managers should be aware that employees with work-life balance problems could experience a reduction in their emotional well-being and also suffer from health problems. The latter, in turn, could increase turnover intention. Finally, we recommend that organisations pay special attention to female managers. This group experiences greater difficulty in achieving work-life balance, compared to other groups.

Among the possible limitations of this study would be that the sample used belongs to the Spanish service sector; i.e., one country and one sector. Although the findings obtained are significant, this sample could suffer a particular bias that would limit the generalisation of the results at a global level and for other sectors. In this regard, a future line of work to overcome this limitation would be to replicate this study in other industries and countries. This would allow us to overcome the bias and compare different sectoral, cultural, economic and socio-demographic contexts.

Comparison between groups considering hierarchy could also be a limitation of this work as the number of managers surveyed was much lower than the number of employees. We recommend expanding the sample of managers in future studies to allow confirmation of the results obtained here but comparing groups of similar size.

Finally, we suggest another future line of research for analysis with, on the one hand, assessment of what the role of WFC could be in the theoretical model proposed in this paper and, on the other hand, assessment of whether there is a significant relationship between the existence and accessibility of WFPs.

## Figures and Tables

**Table 1:** Measurement model. Discriminant validity. HTMT ratio (Heterotrait-Monotrait ratio).

	Existence	Accessibility	Emotional Well-Being	Physical Well-Being	Turnover Intention
Existence					
Accessibility	0.208				
Emotional well-being	0.325	0.409			
Physical well-being	0.293	0.308	0.703		
Turnover intention	0.282	0.336	0.633	0.497	

**Table 2:** Results of the measurement model.

Construction/Indicators	Weight	Composite Reliability	ρA	AVE
*WFPs—existence (Composite, Mode A)*		0.857	0.865	0.550
Your organisation offers them	0.810			
Your organisation reports on them	0.834			
Know what they consist of	0.734			
You have ever used them	0.546			
Know employees who have used them	0.749			
*WFPs—accessibility (Composite, Mode A)*		0.912	0.902	0.512
There is an unwritten rule in my job that an employee cannot attend to family or personal needs during work hours	0.617			
At my workplace, employees who put their family or personal needs ahead of their jobs are not well regarded	0.752			
If you have a problem combining your work responsibilities with family and personal ones, the attitude in my work is: It was your decision, now accept the consequences	0.772			
In my workplace, employees have to choose between advancing their careers or devoting their attention to their personal or family lives	0.731			
In my workplace, employees who apply for permits or licenses for family or personal reasons, or who agree to different schedules for personal or family needs, are less likely to advance in their jobs or careers	0.773			
There would be negative consequences regarding advancing my professional career	0.824			
There would be negative consequences regarding my current or future income	0.687			
My superior would not support it	0.687			
My colleagues would not support it	0.481			
They would make me appear to be less committed to my job or career	0.771			
*Emotion-based well-being (Composite, Mode A)*		0.955	0.954	0.590
Tense	0.773			
Uneasy	0.788			
Preoccupied	0.761			
Calm	0.731			
Content	0.722			
Relaxed	0.708			
Depressed	0.812			
Sad	0.830			
Unhappy	0.794			
Cheerful	0.769			
Enthusiastic	0.666			
Optimistic	0.729			
Angry	0.784			
Annoyed	0.798			
Irritated	0.831			
*Physical well-being (Composite, Mode A)*		0.948	0.940	0.671
Annoying trembling of my hands	0.766			
Shortness of breath when not working physically or hard	0.863			
Pounding heart	0.894			
Faster-than-normal pounding of my heart	0.888			
Sweat on my hands, feeling wet and sticky	0.796			
Momentary dizziness	0.810			
Stomach ache or upset	0.826			
Loss of appetite	0.777			
Problems with sleeping at night	0.738			
*Turnover intention (Composite, Mode A)*		0.898	0.859	0.688
I will probably actively look for another job soon	0.789			
I often think about resigning	0.894			
It would not take much to make me resign	0.863			
There is not too much to be gained by sticking with the organisation indefinitely	0.764			

Note: ρA: Dijkstra-Henseler’s rho, AVE: average variance extracted.

**Table 3:** Direct effects. Hypotheses.

	Direct Effect	*p*-Value	t-Value	Confidence Interval	Supported	Explained Variance	f²
*Turnover intention* *(R² = 0.252)*							
H1(-): Existence	−0.139	0.000	3.538	(−0.205; −0.076)	Yes	3.70%	0.024
H2(-): Accessibility	−0.173	0.000	4.267	(−0.240; −0.105)	Yes	5.28%	0.036
H6(-): Physical well-being	−0.363	0.000	9.268	(−0.429; −0.299)	Yes	16.26%	0.154
*Emotional well-being* *(R² = 0.196)*							
H3(+): Existence	0.229	0.000	6.009	(0.169; 0.294)	Yes	6.76%	0.063
H4(+): Accessibility	0.336	0.000	8.633	(0.274; 0.402)	Yes	12.80%	0.135
*Physical well-being* *(R² = 0.464)*							
H5(+): Emotional well-being	0.681	0.000	32.324	(0.647; 0.716)	Yes	46.38%	0.866

**Table 4:** Indirect effects. Mediation.

	Indirect Effect	*p*-Value	t-Value	Confidence Interval	Supported
Existence—Emotional well-being Physical well-being—Turnover intention	−0.056	0.000	4.767	(−0.077; −0.039)	Yes
Accessibility—Emotional well-being—Physical well-being—Turnover intention	−0.083	0.000	5.930	(−0.108; −0.062)	Yes
Existence—Emotional well-being—Physical well-being	0.156	0.000	5.785	(0.114; 0.203)	Yes
Accessibility—Emotional well-being—Physical well-being	0.229	0.000	8.160	(0.185; 0.277)	Yes
Emotional well-being—Physical well-being—Turnover intention	−0.247	0.000	8.231	(−0.299; −0.200)	Yes

**Table 5:** Results of the measurement invariance assessment (MICOM).

	Step 1	Step 2			Step 3a				Step 3b				
	Configuration Invariance	Composite Invariance			Equality of Variances				Equal Averages				
Groups/Construct		Original Correlation	5%	Supported Partial Measure Invariance	Difference between Original Variances	2.5%	97.5%	Equal	Difference between Original Means	2.5%	97.5%	Equal	Supported Total Measure Invariance
*Female managers-female employees*													
Existence	Yes	0.807	0.441	Yes	−0.080	−0.511	0.354	Yes	0.253	−0.354	0.367	Yes	Yes
Accessibility	Yes	0.985	0.787	Yes	−0.165	−0.663	0.455	Yes	−0.241	−0.349	0.360	Yes	Yes
Emotional well-being	Yes	0.995	0.993	Yes	−0.139	−0.484	0.335	Yes	−0.323	−0.351	0.356	Yes	Yes
Physical well-being	Yes	0.999	0.996	Yes	−0.280	−0.606	0.358	Yes	−0.383	−0.358	0.348	No	No
Turnover intention	Yes	0.998	0.987	Yes	−0.086	−0.463	0.289	Yes	0.083	−0.378	0.334	Yes	Yes
*Female managers-male managers*													
Existence	Yes	0.858	0.602	Yes	−0.278	−0.423	0.387	Yes	0.404	−0.439	0.420	Yes	Yes
Accessibility	Yes	0.991	0.961	Yes	−0.206	−0.642	0.563	Yes	−0.432	−0.407	0.439	No	No
Emotional well-being	Yes	0.988	0.991	No	0.127	−0.681	0.641	Yes	−0.496	−0.407	0.402	No	No
Physical well-being	Yes	0.997	0.987	Yes	0.135	−0.537	0.479	Yes	−0.328	−0.416	0.422	Yes	Yes
Turnover intention	Yes	0.999	0.989	Yes	0.152	−0.445	0.403	Yes	0.384	−0.446	0.415	Yes	Yes
*Female managers-male employees*													
Existence	Yes	0.910	0.616	Yes	−0.241	−0.481	0.347	Yes	0.404	−0.352	0.353	No	No
Accessibility	Yes	0.997	0.942	Yes	−0.396	−0.542	0.423	Yes	−0.522	−0.338	0.357	No	No
Emotional well-being	Yes	0.998	0.995	Yes	−0.201	−0.501	0.335	Yes	−0.397	−0.365	0.370	No	No
Physical well-being	Yes	0.999	0.996	Yes	−0.031	−0.677	0.481	Yes	−0.602	−0.363	0.346	No	No
Turnover intention	Yes	0.997	0.973	Yes	0.026	−0.460	0.311	Yes	0.134	−0.349	0.379	Yes	Yes
*Female employees-male managers*													
Existence	Yes	0.904	0.858	Yes	−0.136	−0.282	0.373	Yes	0.053	−0.301	0.271	Yes	Yes
Accessibility	Yes	0.989	0.939	Yes	−0.051	−0.421	0.446	Yes	−0.220	−0.311	0.264	Yes	Yes
Emotional well-being	Yes	0.998	0.995	Yes	0.280	−0.331	0.387	Yes	−0.135	−0.295	0.278	Yes	Yes
Physical well-being	Yes	0.999	0.998	Yes	0.432	−0.360	0.440	Yes	0.107	−0.277	0.315	Yes	Yes
Turnover intention	Yes	0.998	0.992	Yes	0.237	−0.282	0.356	Yes	0.266	−0.282	0.288	Yes	Yes
*Female employees-male employees*													
Existence	Yes	0.966	0.957	Yes	−0.071	−0.196	0.206	Yes	0.121	−0.184	0.181	Yes	Yes
Accessibility	Yes	0.987	0.991	No	−0.235	−0.254	0.244	Yes	−0.337	−0.178	0.187	No	No
Emotional well-being	Yes	1.000	0.999	Yes	−0.062	−0.224	0.220	Yes	−0.079	−0.178	0.177	Yes	Yes
Physical well-being	Yes	1.000	0.999	Yes	0.252	−0.301	0.293	Yes	−0.167	−0.188	0.182	Yes	Yes
Turnover intention	Yes	0.997	0.996	Yes	0.134	−0.177	0.186	Yes	0.029	−0.170	0.175	Yes	Yes
*Male managers-male employees*													
Existence	Yes	0.980	0.915	Yes	0.046	−0.334	0.252	Yes	0.057	−0.307	0.285	Yes	Yes
Accessibility	Yes	0.996	0.973	Yes	−0.201	−0.439	0.355	Yes	−0.142	−0.314	0.304	Yes	Yes
Emotional well-being	Yes	0.997	0.996	Yes	−0.347	−0.446	0.333	Yes	0.047	−0.307	0.279	Yes	Yes
Physical well-being	Yes	0.999	0.998	Yes	−0.174	−0.540	0.421	Yes	−0.297	−0.323	0.296	Yes	Yes
Turnover intention	Yes	1.000	0.985	Yes	−0.119	−0.397	0.282	Yes	−0.235	−0.287	0.290	Yes	Yes

**Table 6:** Multi-group analysis based on the permutation test.

Groups/Direct Effects	Group 1			Group 2			Permutation	Significance
	R²	Direct Effect	*p*-Value	R²	Direct Effect	*p*-Value	*p*-Value	
*Female managers-female employees*								
Turnover intention	0.441			0.302				
Existence		−0.214	0.136		−0.136	0.012	0.694	No
Accessibility		−0.159	0.150		−0.244	0.000	0.633	No
Physical well-being		−0.602	0.000		−0.372	0.000	0.192	No
Emotional well-being	0.249			0.192				
Existence		−0.276	0.211		0.309	0.000	0.013	Yes
Accessibility		0.347	0.009		0.240	0.000	0.528	No
Physical well-being	0.610			0.474				
Emotional well-being		0.781	0.000		0.689	0.000	0.337	No
*Female managers-male employees*								
Turnover intention	0.441			0.229				
Existence		−0.214	0.136		−0.119	0.030	0.644	No
Accessibility		−0.159	0.150		−0.153	0.015	0.982	No
Physical well-being		−0.602	0.000		−0.352	0.000	0.208	No
Emotional well-being	0.249			0.219				
Existence		−0.276	0.211		0.217	0.000	0.018	Yes
Accessibility		0.347	0.009		0.362	0.000	0.932	No
Physical well-being	0.610			0.479				
Emotional well-being		0.781	0.000		0.692	0.000	0.231	No
*Female employees-male managers*								
Turnover intention	0.302			0.259				
Existence		−0.136	0.012		−0.252	0.050	0.436	No
Accessibility		−0.244	0.000		−0.085	0.258	0.193	No
Physical well-being		−0.372	0.000		−0.321	0.031	0.708	No
Emotional well-being	0.192			0.394				
Existence		0.309	0.000		0.342	0.000	0.782	No
Accessibility		0.240	0.000		0.469	0.000	0.080	No
Physical well-being	0.474			0.355				
Emotional well-being		0.689	0.000		0.596	0.000	0.253	No
*Male managers-male employees*								
Turnover intention	0.259			0.229				
Existence		−0.252	0.050		−0.119	0.030	0.430	No
Accessibility		−0.085	0.258		−0.153	0.015	0.710	No
Physical well-being		−0.321	0.031		−0.352	0.000	0.846	No
Emotional well-being	0.394			0.219				
Existence		0.342	0.000		0.217	0.000	0.379	No
Accessibility		0.469	0.000		0.362	0.000	0.443	No
Physical well-being	0.355			0.479				
Emotional well-being		0.596	0.000		0.692	0.000	0.174	No

## Data Availability

The data presented in this study are available on request from the corresponding author. The data are not publicly available due to the fact that access to them requires express and unique approval for each occasion.
